# Effects of processing and storage conditions of cocoyam strips on the quality of fries

**DOI:** 10.1002/fsn3.358

**Published:** 2016-03-29

**Authors:** Oyindamola Oguntowo, Adewale O. Obadina, Olajide P. Sobukola, Mojisola O. Adegunwa

**Affiliations:** ^1^ Department of Food Science and TechnologyFederal University of AgricultureP.M.B. 2240AbeokutaNigeria; ^2^ Department of Hospitality and TourismFederal University of AgricultureAbeokutaNigeria

**Keywords:** Cocoyam, French fries, frozen, frying, storage

## Abstract

The effects of blanching time and temperature on the sensory and textural properties of frozen cocoyam strips were studied for cocoyam varieties. The most preferred variety after sensory evaluation was blanched at 90°C for 5 min, reproduced, and frozen at a temperature of −18°C for storage studies over a period of 12 weeks with Irish potato as control. Sensory evaluation and instrumental texture analysis of frozen fried samples were conducted at 3 weeks intervals for 12 weeks. Sensory evaluation during storage showed no significant difference (*P* < 0.05) in taste, aroma, and mouth feel attributes between control and cocoyam fries. The sensory score for taste, sogginess, and mouth feel increased while those for aroma and color decreased in comparison with the control fries over storage. The texture increased during storage and for control fries. There was a significant negative correlation between sogginess, hardness, and dry matter, respectively.

## Introduction

Cocoyams (*Colocasia esculenta* and *Xanthosoma sagittifolium*) are herbaceous perennial plants belonging to the family araceae and are grown primarily for their edible roots. They are among the tuberous roots well adapted to most agro‐ecological zones in Nigeria. The corm, cormels, and leaves are important sources of carbohydrates for human consumption and animal feed, and a good source of income for farmers in Asia, Africa, and Latin America. They contain digestible starch, good quality protein, vitamin C, thiamine, riboflavin, niacin, and high scores of essential amino acids (Lewu and Adebola 2010). In spite of all the health benefits cocoyams are neglected and they face a major problem, which is high susceptibility to physical damage, during harvesting leading to high postharvest losses. It is grossly underutilized as much of it is limited to direct consumption through boiling of the tuber, frying in oil, or pounding into fufu (a dumpling similar to pounded yam), and eaten with soup and also used as a soup thicker in some parts of the world (Ejor et al. 2013).

Although some work has been done in recent times on other ways of utilization such as the use of its starch industrially, use of the tuber in the brewing industry, and use of cocoyam flour as a composite with other cereals for snack production (Owusu‐Darko et al. 2014; Delpeuch et al. 1975; Addisu et al. 2014); not much has been done to extend its shelflife by value addition or in new product development.

A comparative assessment of cocoyam and potato was carried out by Lewu and Adebola (2010) and it was concluded that commercially available cocoyam and potato have very close nutritional values. Cocoyam was compared favorably with potato and even excelled in some nutrients and minerals such as iron, calcium, and sodium, thus cocoyam consumption should be encouraged and popularized as an additional tuber crop (Lewu and Adebola 2010). French fries which have been defined by Wikipedia as elongated thinly cut slices of deep fried potato have in recent times gained popularity in quick service restaurants globally (Quansah and Saalia 2010). Irish potatoes which are used for fries are not planted in most of the developing countries making it an expensive food. The use of cocoyam to produce fries of similar nature will not only make them more affordable, but will also enhance its distribution network, while simultaneously overcoming the perishability of the crop, and add economic value.

In view of these reasons, this research work was carried out to determine the performance of cocoyam as an alternative to potato in French fries production. The most suitable cocoyam variety and the best pretreatment (blanching time and temperature) that will produce acceptable fries were determined. Storage studies on the cocoyam fries from selected samples of cocoyam frozen at −10°C at different intervals was also carried out.

## Materials and Methods

### Materials

Two varieties of cocoyam (*Xanthosoma sagittifolium* and *Colocasia esculenta*) and Irish potato (*Solanum tuberosum*) were obtained from Ile Epo and Mushin markets in Lagos State, Nigeria.

### Preparation of steam blanched cocoyam fries

The two varieties of cocoyam tubers were washed, peeled manually, and sliced into strips of about 1 × 1 cm in cross section and 6–7 cm in length using a potato slicer. The strips were soaked in 0.5% ascorbic acid solution for 15 min to prevent enzymic browning. The strips were then blanched in hot water at various temperatures (70°C, 80°C, and 90°C) and at different time intervals (5, 10, and 15 min). They were cooled at ambient temperature and fried at 160°C for 4 min using the methods of Quansah and Saalia (2010) and Aseidu‐Larbi (2010).

### Experimental design

A 3 × 3 full factorial design was used for the research work. The factors were cocoyam varieties (*Xanthosoma sagittifolium* and *Colocasia esculenta*), blanching time (5, 10, and 15 min), and blanching temperature (70°C, 80°C, and 90°C).

### Frying of steam blanched cocoyam strips

Freshly blanched cocoyam strips (300 g) were fried in deep fryer with vegetable oil at 160°C for 4 min, drained, and served to panelists for sensory evaluation.

### Determination of the effect of steam blanching on sensory acceptability of cocoyam French fries

Samples (*n* = 18) were picked from the two varieties, steam blanched at different time intervals (5, 10, and 15 min), and varying temperatures (70°C, 80°C, and 90°C), and subsequently fried. These were coded and presented to consumer panelists for the evaluation of color, flavor (taste and aroma), texture, and overall acceptability of the samples.

### Consumer acceptability test

Sensory evaluation was carried out using a panel of 30 untrained consumer panelists who are familiar with French fries to determine the presence of detectable differences in the color, taste, aroma, crispiness, sogginess, and overall acceptability of the fries from different treatments. Traditional French fries were used as control. The samples were coded and scored on a 7‐point hedonic scale (Quansah and Saalia 2010). The most preferred sample from the two varieties and pretreatments was selected and packaged for frozen storage and studies. This was stored in the freezer at −18°C for 12 weeks and evaluated (microbial analysis, sensory evaluation, and texture analysis) at an interval of 3 weeks.

### Sensory evaluation

Trained panelists were used in carrying out the sensory tests. Paired comparison test, quantitative descriptive test, and consumer acceptability were carried out on the samples.

### Training of panelists

Descriptor generation sessions were carried out during which panelist tasted the cocoyam fries and generated terms to describe their attributes. Once a conclusion was reached on each attribute, definitions for each attribute were established by the panel leader. The training involved two sessions in which the panelists were familiarized with different attribute note and also trained for performing the quantitative descriptive analysis test (Stone and Sidel 1993; Murray 2001).

### Paired comparison test and descriptive sensory profiling

Blanched frozen pink cocoyam samples and Irish potato (reference) were subjected to paired comparison test to detect the differences between the parameters (color, taste, aroma, mouth feel, and sogginess) by 15 trained panelists for a period of 12 weeks at 3 weeks intervals. Testing was performed in a sensory laboratory with individual booth under fluorescent lighting equal to daylight. Two pieces each of pink cocoyam fries and Irish potato were coded and served, while water was used as palate cleansing material in between the samples (Chaiyakul et al. 2008). The score card used for descriptive analysis consisted of 15 cm quantitative descriptive analysis scale and the panelists were asked to indicate their opinion by marking on the 15 cm line scaling (Meilgaard et al. 1999).

### Consumer acceptability test

Also a structured 9‐point hedonic scale (where 1 = like extremely and 9 =  dislike extremely) was used to determine the acceptability of pink cocoyam among 120 panelists who are regular consumers of French fries. Their responses were made on score sheets which had been designed in line with the test procedures according to Iwe (2002).

### Instrumental texture analysis

Texture profile analysis for the strips was determined using a texture analyzer (Model TA‐XT2i, stable micro system, Haslemere, UK). The samples were supported on two parallel edges so that the load could be applied centrally using a support span of 30 mm at a speed of 20 mm/min. The force (N) at the fracture point highest value in the plot corresponded to the force at peak which is also the resistance to break. This was carried out on the steam blanched French fried cocoyam on the first day and on the third, sixth, ninth, and twelfth week.(DA Silver and Moreira 2008)

### Data analysis

All procedures were carried out in triplicates and data collected from the study were subjected to analysis of variance (ANOVA). Differences among means were separated using Duncan's multiple range test and significances were accepted at 5% level (*P* < 0.05) (Duncan 1955). Pearson's correlation coefficient between texture and weight loss of the preserved and stored samples was calculated and the statistical software SAS 9.0 (2008) (SAS Institute, Inc., Cary, NC, USA) was used.

## Results and Discussion

Blanching at a temperature of 90°C for 15 min produced the most acceptable color for the white cocoyam variety with a score of 2.04, while the most acceptable color for the pink cocoyam variety was obtained at blanching temperature and time of 80°C for 10 min, respectively. Table 1 shows there was a significant difference between the pink and the white varieties, as it had mean values of 2.52. The sensory evaluation results showed significant difference in the consumer preference for color (*P* < 0.05) as shown in Table 1. The white variety which was most preferred produced a golden brown color after frying. The results show that the first five most acceptable fries were the white variety as shown in Table 1 and there were no significant difference in the acceptability, while the other pink samples had significant difference at *P* < 0.05. This confirms the fact that consumers prefer French fries which are golden brown in color (Aseidu‐Larbi 2010). The differences in color are due to varietal differences.

**Table 1:** Preference order for color attribute of cocoyam fries

Cocoyam variety	Blanching time (min)	Blanching temperature (°C)	Mean	Order of ranks
White	15	90	2.04^bc^	1st
White	10	70	2.12^bc^	2nd
White	5	70	2.28^bc^	3rd
White	5	90	2.32^bc^	4th
White	10	90	2.36^bc^	5th
White	10	80	2.40^bc^	6th
Pink	10	80	2.52^ab^	7th
Pink	15	70	2.52^abc^	8th
White	5	80	2.56^abc^	9th
Pink	15	90	2.60^ab^	10th
Pink	15	80	2.60^ab^	11th
Pink	10	90	2.63^a^	12th
Pink	5	90	2.64^a^	13th
Pink	5	70	2.68^ab^	14th
Pink	5	80	2.68^ab^	15th
Pink	10	70	2.80^ab^	16th
White	15	70	2.88^a^	17th
White	15	80	2.92^a^	18th

Figures with the different subscript across columns shows significant difference (p<0.05) level.

The flavor was assessed using a combination of taste and aroma. The differences in flavor acceptability among the samples were significant (*P* < 0.05) as shown in Table 2. The pink variety blanched at 90°C for 5 min was most accepted, with the least accepted being the white variety blanched at 80°C for 15 min. They both scored 1.84 and 2.92, respectively, as shown in Table 2. The most preferred three samples in terms of flavor were the pink cocoyam varieties blanched at 90°C for 5 and 10 min and the one blanched at 70°C for 5 min. These differences could be attributed to the different varieties of the cocoyam and the different blanching temperature and time used in processing. As stated in the Grading Manual for Frozen French Fries, the flavor of French fried potatoes is affected by the conditions of the potatoes with respect to sugar, sunburn, the condition of the fat or oil used, and to a certain extent by the variety of the potatoes, the type of soil, and climatic conditions (United States Department of Agriculture [USDA] 1994)

**Table 2:** Preference ranking for flavor attribute of cocoyam fries

Cocoyam variety	Blanching time (min)	Blanching temperature (°C)	Mean	Order of ranks
Pink	5	90	1.84^b^	1st
Pink	10	90	1.96^b^	2nd
Pink	5	70	2.04^b^	3rd
White	15	90	2.12^b^	4th
Pink	10	80	2.20^ab^	5th
White	5	90	2.28^ab^	6th
Pink	15	90	2.36^ab^	7th
Pink	15	80	2.40^ab^	8th
Pink	5	80	2.48^ab^	9th
White	10	90	2.68^ab^	10th
White	10	70	2.72^ab^	11th
Pink	10	70	2.72^a^	12th
White	5	80	2.76^ab^	13th
Pink	15	70	2.84^a^	14th
White	15	70	2.84^a^	15th
White	5	70	2.88^a^	16th
White	10	80	2.92^a^	17th
White	15	80	2.92^a^	18th

Mouth feel is the degree of roughness or smoothness of a food sample in the mouth. The differences in mouth feel acceptability among the samples were significant (*P* < 0.05). The pink variety blanched at 90°C for 5 min was most accepted, with the least accepted being the pink variety blanched at 70°C for 10 min. They both scored 2.04 and 3.04, respectively, as shown in Table 3. Differences in mouth feel can be attributed to differences in dry matter content. High dry matter is associated with fine structure, dense mouth feel, and quality (Aseidu‐Larbi 2010). This is in harmony with the assertion by Kabira and Berga Lemago (2003) that the dry matter partly determines the texture and oiliness of the finished product. Potatoes with dry matter content of 20–24% are ideal for making French fries since some dry matter is lost during peeling, trimming, slicing, and blanching. The higher the initial dry matter content, the higher the amount which remains after frying (Kabira and Berga Lemago 2003). The first three most acceptable samples was the pink variety, while the fourth in line was the white variety. This confirms that the higher the initial dry matter content, the higher the amount which remains after frying as the pink variety has a higher dry matter than the white variety.

**Table 3:** Preference order of flavor attribute of cocoyam fries

Cocoyam variety	Blanching time (min)	Blanching temperature (°C)	Mean	Order of ranks
Pink	5	90	2.04^b^	1st
Pink	5	70	2.16^b^	2nd
Pink	10	80	2.24^b^	3rd
White	15	90	2.28^b^	4th
Pink	5	80	2.40^ab^	5th
Pink	10	90	2.44^ab^	6th
Pink	15	90	2.48^ab^	7th
Pink	15	80	2.64^ab^	8th
White	10	70	2.76^ab^	9th
White	5	90	2.76^ab^	10th
White	5	70	2.80^ab^	11th
White	15	80	2.84^ab^	12th
White	10	90	2.92^a^	13th
Pink	15	70	2.92^a^	14th
White	15	70	2.96^a^	15th
White	5	80	2.96^a^	16th
White	10	80	2.96^a^	17th
Pink	10	70	3.04^a^	18th

Crispiness is defined as the force and noise with which a product breaks or fractures rather than deforms when chewed with the molar teeth (Meilgaard et al. 1999; King Maria 2005). It is associated with crunchiness, crackling freshness, brittleness, snapping, and sound emission during eating (Szczesniak 1988). There were significant differences between the crispiness of fried chips from the different varieties and treatments (*P* < 0.05). The pink variety blanched at 70°C for 5 min at a mean score of 2.32 was most accepted followed by the pink variety blanched at 90°C for 5 min scoring 2.44. The least accepted was the white variety blanched at 80°C for 10 min with a mean score of 3.40 as shown in Table 4.

**Table 4:** Preference order of crispiness attribute of cocoyam fries

Cocoyam variety	Blanching time (min)	Blanching temperature (°C)	Mean	Order of ranks
Pink	5	70	2.32^b^	1st
Pink	5	90	2.44^b^	2nd
Pink	15	70	2.52^b^	3rd
Pink	5	80	2.56^b^	4th
Pink	15	80	2.64^a^	5th
Pink	10	90	2.76^a^	6th
Pink	10	70	2.76^a^	7th
White	10	70	2.76^a^	8th
Pink	10	80	2.80^a^	9th
White	15	70	2.88^a^	10th
White	5	80	2.92^a^	11th
White	5	70	2.92^a^	12th
White	5	90	2.92^a^	13th
Pink	15	90	3.04^a^	14th
White	15	90	3.04^a^	15th
White	15	80	3.32^a^	16th
White	10	90	3.36^ab^	17th
White	10	80	3.40^a^	18th

The differences in sogginess among the samples were significant (*P* < 0.05). The white variety blanched at 70°C for 10 min and 15 min was the most accepted and had no significant difference as they both scored 2.04 as shown in the Table 5. The least accepted fries were the white variety blanched at 90°C for 10 min with a score of 2.76. There was no difference in the sogginess parameter between the white cocoyam variety blanched at 70°C for 10 and 15 min, 80°C for 5 min, and 90°C for 5 and 15 min and the pink cocoyam variety blanched at 70°C for 5, 10, and 15 min, 80°C for 5 and 10 min, and 90°C for 5 and 10 min. Sogginess as the name implies refers to a wet pasty or mushy condition either loaded with water or oil. This may be induced by frying at low temperatures (grading manual) or under cooking. Utilizing the proper management techniques and a high stability oil will allow for consistency in production (Gelski 2011; Elevina 2000).

**Table 5:** Preference order for sogginess attribute of cocoyam fries

Cocoyam variety	Blanching time (min)	Blanching temperature (°C)	Mean	Order of ranks
White	10	70	2.04^bc^	1st
White	15	70	2.04^bc^	2nd
White	5	80	2.20^bc^	3rd
Pink	15	70	2.20^bc^	4th
Pink	5	90	2.24^bc^	5th
Pink	5	70	2.24^bc^	6th
White	5	90	2.28^bc^	7th
White	15	90	2.36^bc^	8th
Pink	10	70	2.36^bc^	9th
Pink	5	80	2.44^bc^	10th
Pink	10	80	2.44^bc^	11th
Pink	10	90	2.48^bc^	12th
Pink	15	80	2.52^b^	13th
White	15	80	2.56^b^	14th
White	10	80	2.64^ab^	15th
Pink	15	90	2.72^a^	16th
White	5	70	2.72^a^	17th
White	10	90	2.76^a^	18th

The results obtained showed that the white variety blanched at 90°C for 15 min had the best color acceptability but was not preferred for other parameters compared to the pink variety blanched at 90°C for 5 min which ranked 12th. The pink variety blanched at 90°C for 5 min was most preferred in terms of flavor and mouth feel. With relation to crispiness it ranked second scoring 2.44 and ranked fifth in terms of sogginess with a score of 2.24. Thus, based on the overall acceptability, the pink variety blanched at 90°C for 5 min was selected for storage studies. The overall accepted pink cocoyam strips were stored for 12 weeks at a temperature of −18°C and analyzed for texture and sensory properties at the interval of 3 weeks after frying. Also, the texture analyzer was used to measure the hardness of the fries after deep frying. Hardness or force at peak (the peak force of the first compression of the product) increased slightly throughout the duration of storage with average values ranging from 2.95 N at week 0 to 4.67 N at the end of the 12 weeks of storage for cocoyam while that of potato ranged from 1.30 to 2.45 N, as shown in Table 6.

**Table 6:** Hardness of *Xanthosoma sagittifolium* and Irish potato before and during storage

Interval (weeks)	Force at peak (N)	
	Cocoyam	Potato
0	2.95	1.30
3	3.10	1.65
6	4.20	1.73
9	4.63	2.40
12	4.67	2.45

Texture is considered one of the most important quality aspects of French fries. There is a clear distinction between the interior and the exterior. The interior has to be soft and mealy, whereas the exterior consists of a crispy crust which is formed during frying and generally has a thickness of about 1 mm. Blanching affects the texture of plant tissues and that of French fries as it is responsible for starch gelatinization which reduces oil uptake during frying and texture improvement according to Loon (2005). Blanching and frying increases the dry matter content of French fries due to losses of nonfiber substances (Aseidu‐Larbi 2010). According to Aseidu‐Larbi (2010), blanching not only preserves texture in the freezer, but also the water is lost or ice crystals evaporate from the surface of a food product making the surface dry and tough. The loss of water from the product contributes to an increase in dry matter content thereby increasing the hardness of the final product. The technological parameters for blanching also affect the texture of plant tissues and consequently the texture of French fries (Alvarez and Canet 1999)

Trained panelists analyzed the fried cocoyam chips using five parameters which were color, taste, aroma, sogginess, and mouth feel. Table 7 shows the results of the sensory evaluation on the cocoyam fries in storage for weeks 3 to 12. Yam flavor was used as the reference point in evaluating the aroma of the fries. In terms of taste there were no significant changes in the taste acceptability of the cocoyam and potato over the 12 weeks of storage. The taste mean values for cocoyam ranged from 2.86 on week 0 to 2.72 on week 12, while potato ranged from 2.90 to 2.76 throughout weeks 0 to 12. The tastes of cocoyam fries were more acceptable than that of potato as shown in Table 7. These differences could be attributed to the difference varieties of the cocoyam and the different blanching temperature and time used in processing.

**Table 7:** Paired comparison between cocoyam and Irish potato

Parameters	Weeks									
	0		3		6		9		12	
	Potato	Cocoyam	Potato	Cocoyam	Potato	Cocoyam	Potato	Cocoyam	Potato	Cocoyam
Taste	2.90^a^	2.86^a^	2.87^a^	2.80^a^	2.85^a^	2.77^a^	2.80^a^	2.75^a^	2.76^a^	2.72^a^
Aroma	2.20^a^	2.12^a^	2.47^a^	2.53^a^	2.47^a^	2.53^a^	2.56^a^	2.60^a^	2.66^a^	2.61^a^
Color	1.93^a^	2.13^a^	2.06^a^	2.21^a^	2.07^a^	2.36^b^	2.27^a^	3.12^b^	2.53^a^	3.40^b^
Sogginess	3.57^a^	3.47^a^	3.50^a^	3.58^a^	3.47^b^	3.07^a^	3.25^a^	2.70^b^	2.47^a^	2.53^a^
Mouth feel	1.94^a^	2.07^a^	2.47^a^	2.57^b^	2.73^a^	2.85^a^	3.00^a^	3.07^a^	3.07^a^	3.12^a^

Values are means of triplicate determination. Mean values with different superscript alphabets within the same column are significantly different (*P* < 0.05).

The mean values for cocoyam increased slightly from 3.47 before storage to 3.58 after 3 weeks of storage and reduced gradually to 2.53 by week 12 in freeze storage. While that of potato was 3.57 before storage, during storage it ranged from 3.50 to 2.47 as shown in Table 7. Also Table 7 shows that sogginess is negatively correlated with hardness. For both products, *r* = −0.361 and −0.689, respectively. Thus, the harder the product, the less oil absorbed and the less soggy the product is. Sogginess is also negatively correlated with dry matter with *r* = −0.809. This implies that the higher the dry matter of the fresh tuber, the less soggy the end product is. This also explains why the mouth feel of potato was more acceptable during storage compared to potato during storage, though there was no significant difference at *P* < 0.05.

There were no significant differences in the color acceptability of both cocoyam and potato (*P* < 0.05) before storage and during storage up till week 12 at *P* < 0.05. The color of potato was more acceptable compared to cocoyam. Before storage, potato and cocoyam had mean values of 1.93 and 2.13, respectively. During storage the mean values of potato ranged from 2.06 to 2.53, while that of cocoyam ranged from 2.21 to 3.40 as shown in the table. Good fries color is light cream to golden brown, while desirable textural characteristics are a crisp outer crust and a soft mealy interior. (Talburt et al. 1987; Lisinska and Leszcynski 1989). Color quality of French fries is mainly determined by the extent of Millard browning reaction which occurs at high processing temperatures between reducing sugars and free amino acids (Roe et al. 1990; Akinlua et al. 2013). The exact color of good quality French fries varies because of varietal difference, physical difference, type of fat used, areas of production, and color induced by the frying process (Grading Manual 1994). This explains why fries from *Colocasia esculenta* being white in color produced fries whose color, light golden brown, was similar to fries produced from Irish potato and thus were mostly preferred than the fries from *Xanthosoma sagittifolium* which were dark golden brown in color.

In terms of mouth feel, the cocoyam was more acceptable than that of potato. Before storage, there was no significant difference at *P* < 0.05 as cocoyam had a mean value of 1.94, while potato had a mean value of 2.07. At week 3 of storage, significant difference was observed as cocoyam had a mean value of 2.57 and potato had a mean value of 2.47, but there were no differences observed during storage from weeks 6 to 12 as shown in the table. There were only slight increases in the mouth feel with mean values ranging from 2.85 to 3.12 for cocoyam and 2.73 to 3.07 for potato. Differences in mouth feel can be attributed to differences in dry matter content. High dry matter is associated with fine structure, dense mouth feel, and quality (Aseidu‐Larbi 2010). This is in harmony with the assertion by Kabira and Berga Lemago (2003) (Adegunwa et al. 2011) that the dry matter partly determines the texture and oiliness of the finished product. Potatoes with dry matter content of 20–24% are ideal for making French fries since some dry matter is lost during peeling, trimming, slicing, and blanching. The higher the initial dry matter content, the higher the amount which remains after frying (Kabira and Berga Lemago 2003). The first three most acceptable samples was the pink variety, *Xanthosoma sagittifolium*, while the fourth in line was the white variety, *Colocasia esculenta*. This confirms that the higher the initial dry matter content, the higher the amount which remains after frying, as the pink variety has a higher dry matter than the white variety, though the dry matter content of both varieties fall within the desired range.

Although there were no observed significant differences in the aroma attribute (*P* < 0.05) before storage, there were slight increments in the aroma intensity during the 12 weeks of storage. Before storage the mean values of potato was 2.20 and cocoyam was 2.12. During storage the mean values ranged from 2.47 to 2.66 for potato, while that of cocoyam ranged from 2.53 to 2.61. The sensory parameters such as taste, aroma, color, mouth feel, and sogginess had mean values ranging from 7.00 to 6.50, 7.67 to 7.33, 6.17 to 8.19, 9.50 to 7.43, and 6.67 to 4.96, respectively, as shown in the Table 8.

**Table 8:** Descriptive analysis of cocoyam fries

Weeks	Taste	Aroma	Color	Mouth feel	Sogginess
0	7.00^a^	7.67^a^	6.17^a^	9.50^a^	6.67^b^
3	6.43^a^	5.60^a^	6.00^a^	8.17^a^	3.33^a^
6	6.09^a^	7.57^a^	6.27^a^	7.99^a^	7.22^b^
9	6.73^a^	6.67^a^	9.17^a^	10.09^a^	5.10^ab^
12	6.50^a^	7.33^a^	8.19^a^	7.43^a^	4.96^ab^

Values are means of triplicate determination. Mean values with different superscript alphabets within the same column are significantly different (*P* < 0.05). Attribute, descriptor; Taste, sweet; Aroma, moderate yam flavor; Color, dark golden brown; Mouth feel, moderately smooth; Sogginess, slightly soggy.

The results of the consumer acceptability using the pink cocoyam variety, the white cocoyam variety, and Irish Potato show significant differences between the pink cocoyam and Irish potato in terms of taste, color, mouth feel, and sogginess with mean values of 2.21 and 3.00, 3.27 and 1.86, 2.40 and 3.02, and 2.06 and 3.18, respectively. There is no significant difference in terms of aroma as pink cocoyam was rated 2.63 while potato was rated 2.59. In all, cocoyam was readily accepted in terms of all the other parameters except color and aroma for which potato had mean values of 1.86 and 2.59, respectively, unlike cocoyam which scored 3.27 and 2.63, respectively, as shown in Table 9.

**Table 9:** Result of consumer acceptability test

Sample	Taste	Aroma	Color	Mouth feel	Sogginess
White cocoyam	3.09^b^	3.06^b^	3.05^b^	2.94^b^	2.46^a^
Pink cocoyam	2.21^a^	2.63^a^	3.27^b^	2.40^a^	2.06^a^
White yam	3.89^c^	3.47^c^	3.08^b^	3.76^c^	3.15^b^
Irish potato	3.00^b^	2.59^a^	1.86^a^	3.02^b^	3.18^b^

Values are means of triplicate determination. Mean values with different superscript alphabets within the same column are significantly different (*P* < 0.05).

## Conclusion

In conclusion, the best variety for the production of cocoyam fries with similar sensory properties to potato French fries in terms of taste, aroma, mouth feel, and sogginess is the pink variety (*Xanthosoma sagittifolium*) although the color was not ranked low. Blanching at 90°C for 5 min gave the best product in terms of sensory quality, thus this is the best blanching temperature and time for producing acceptable cocoyam fries. It was also observed that at the end of the 12 weeks storage period for the cocoyam strips, the sensory characteristics for the fries produced from it were acceptable.

## Conflict of Interest

None declared.
